# Treatment-Induced Neuropathy of Diabetes: Clinical Insights from a Case Series and Systematic Review

**DOI:** 10.3390/jcm15218760

**Published:** 2026-11-09

**Authors:** Nicu Catalin Draghici, Ruxandra Mosorescu, Cornelia Bala, Dana Mihaela Ciobanu, Hanna Maria Dragoș, Cristina Tecar, Livia Livinț Popa, Dafin Fior Mureșanu

**Affiliations:** 1 Department of Clinical Neurosciences, “Iuliu Hatieganu” University of Medicine and Pharmacy, No. 8 Victor Babeș Street, 400012 Cluj-Napoca, Romania; nicu.draghici@umfcluj.ro (N.C.D.); hanna_dragos2006@yahoo.com (H.M.D.); livia.popa@umfcluj.ro (L.L.P.); dafinm@ssnn.ro (D.F.M.); 2 IMOGEN Institute, Centre of Advanced Research Studies, 400347 Cluj-Napoca, Romania; 3 RoNeuro Institute, Centre for Neurological Research and Diagnostic, 400364 Cluj-Napoca, Romania; 4 NeuroBrain Clinic, Calea Dumbrăvii, nr 46A, 550324 Sibiu, Romania; ruxandrageorgescu03@gmail.com; 5 Department of Diabetes and Nutrition Diseases, Faculty of Medicine, “Iuliu Hatieganu” University of Medicine and Pharmacy, 2–4 Clinicilor Street, 400006 Cluj-Napoca, Romania; 6 Diabetes and Nutrition Diseases Center, Emergency Clinical County Hospital Cluj, 400006 Cluj-Napoca, Romania

**Keywords:** treatment-induced neuropathy of diabetes, diabetic neuropathies, small-fiber neuropathy, glycemic control, case series, systematic review

## Abstract

**Background/Objectives:** Treatment-induced neuropathy of diabetes (TIND) is an under-recognized iatrogenic neuropathy that follows a rapid fall in glycated hemoglobin (HbA1c). We aimed to systematically summarize the published cases of TIND and to compare them with two patients evaluated at our center. **Methods:** We evaluated two patients at our center and systematically reviewed PubMed, Scopus, Web of Science and Google Scholar, following PRISMA 2020, for primary clinical studies of TIND. Data were synthesized descriptively. **Results:** Both patients developed painful neuropathy with autonomic symptoms after HbA1c falls of 15.3 and 6.4 percentage points and improved substantially, but incompletely, over two years. Moreover, the review included 47 publications (219 patients) and painful neuropathy with autonomic dysfunction predominated. In case reports, the median HbA1c fall was 7.3 percentage points, and onset occurred within 8 weeks in 26 of 47 publications. Recovery was complete in 8 and partial in 21 of 32 case reports with documented outcomes. Follow-up data were missing for 136 of 219 patients. **Conclusions:** Published cases show a recurrent temporal association between TIND and rapid HbA1c reduction, but cannot establish causality or prognosis. TIND should be recognized early, and gradual, monitored glycemic correction may be reasonable in high-risk patients.

## 1. Introduction

Diabetes mellitus is complicated by a broad spectrum of neuropathies, whose burden continues to grow worldwide [1,2]. Diabetic neuropathies can have a symmetrical or asymmetrical clinical presentation. Depending on their distribution, they may be symmetric or asymmetric. Symmetric forms are the most common and comprise episodic neuropathies, such as treatment-induced neuropathy of diabetes (TIND) [3,4] and hyperglycemic neuropathy, and persistent neuropathies, such as distal length-dependent polyneuropathy and small-fiber neuropathy [5]. Asymmetrical forms are less frequent and include mononeuropathies, lumbosacral radiculoplexus neuropathy, and cranial and thoracic neuropathies [6]. TIND, formerly referred to as “insulin neuritis,” is an iatrogenic neuropathy that occurs in both type 1 diabetes mellitus (T1DM) and type 2 diabetes mellitus (T2DM) following a rapid improvement in glycemic control, achieved through the administration of insulin, oral hypoglycemic agents, or dietary restriction, as evidenced by a marked decrease in glycated hemoglobin (HbA1c) levels [3,4,7]. It typically presents acutely, 2–8 weeks after the change in glycemic control, manifesting as severe distal neuropathic pain associated with paresthesias and frequent autonomic dysfunction [7,8]. Furthermore, both the risk and the severity of TIND increase with the magnitude and rapidity of the reduction in HbA1c levels [4,7,8]. Neuropathic pain may resolve with stable glycemic control, often over 12–24 months [4]. Moreover, in an 8-year follow-up study of patients with T1DM, neuropathy and microvascular complications improved in those who maintained stable glycemic control, whereas they worsened in those with unstable control [9].

The pathophysiology of TIND is not fully understood. A leading hypothesis points to “relative” hypoglycemia within nerves adapted to chronic hyperglycemia, which leads to a dysfunction in energy-dependent axonal transport [4]. In humans, experimental hypoglycemia has induced tactile hyperalgesia, accompanied by an increase in the levels of pro-inflammatory cytokines [10]. Other proposed mechanisms include arteriovenous shunting with endoneurial ischemia [11,12] or ectopic discharges from regenerating nerve fibers [4], as well as the direct effects of insulin on nociceptive signaling, mediated by insulin-like growth factor 1 [13]. In a rodent model, an abrupt reduction in HbA1c produced nerve conduction slowing and macrophage infiltration of the sciatic nerve [14].

Gibbons and Freeman proposed three diagnostic criteria for TIND: (1) a decrease in HbA1c of at least 2 percentage points per 3 months, applied proportionally over longer intervals; (2) neuropathic pain with acute onset—defined as an increase of at least 3 points on an 11-point Likert scale—and/or autonomic dysfunction developing over the course of 2 weeks, with a severity that prompts the patient to seek medical attention; and (3) the onset of neuropathic pain and/or autonomic symptoms within 8 weeks of the reduction in HbA1c [8,15].

Clinically, pain is typically burning or lancinating, length-dependent and accompanied by allodynia. At the same time, autonomic involvement may include orthostatic intolerance, gastrointestinal dysmotility, sudomotor and erectile dysfunction [4,8]. Because these features overlap with other acute or subacute neuropathies, inflammatory, infectious, metabolic and nutritional causes should be excluded [16,17]. Nerve conduction study (NCS) may be normal when small fibers are predominantly affected, but they are essential for identifying concomitant large-fiber or demyelinating processes. Moreover, skin biopsy and autonomic testing provide objective evidence of small-fiber and autonomic dysfunction [16,17,18,19,20,21].

Despite these proposed criteria, TIND remains under-recognized [4,7]. Its definition, clinical features and dose-dependent association with HbA1c reduction are established, but mainly from single-center cohorts [8,9,22]. Published cases have recently been summarized in a narrative review limited to adults publications from two databases and a systematic review of intensive glycemic treatment discussed TIND only narratively [3,23]. A systematic synthesis of all published pediatric and adult cases, quantifying the HbA1c falls and onset intervals preceding the neuropathy, the reported clinical phenotypes, treatments and outcomes, and the frequency of concurrent microvascular complications, is still lacking. Moreover, no clinical practice guidelines are available for the prevention or management of TIND [15]. Thus, the primary objective of this systematic review was to identify published cases of TIND and to describe their demographic characteristics, type of diabetes, comorbidities and microvascular complications, magnitude and rate of HbA1c reduction, time to symptom onset, clinical phenotype, management and outcome. The secondary objective was to report two patients with TIND evaluated and treated by the authors and to compare their clinical, electrodiagnosis (EDX) and outcome characteristics with those of the published cases.

## 2. Materials and Methods

### 2.1. Case Series

This case series includes two patients who were evaluated, diagnosed, and treated by the authors for TIND, following rapid improvement in glycemic control. Clinical, laboratory, and EDX data were retrospectively collected from medical records. The diagnosis of TIND was based on clinical presentation, the temporal correlation with hyperglycemia correction and the exclusion of other causes of neuropathy. In Case 2, two others potential contributing factors were identified, and the diagnosis is therefore considered less certain. Neuropathic pain was assessed with the Douleur Neuropathique en 4 questions (DN4) [24,25], and neuropathy severity with the Toronto Clinical Neuropathy Score [26], at baseline and at follow-up. EDX studies were performed with Nicolet EDX system (Natus Medical Incorporated, Middleton, WI, USA), with limb temperature maintained above 32 °C. The research was performed in accordance with the standards of the Declaration of Helsinki and approved by the Ethics Committee of the Emergency Clinical County Hospital Cluj-Napoca, Romania (No. 3942/29 January 2026). Written informed consent was obtained from the two patients.

### 2.2. Systematic Review

A systematic literature review was conducted to identify primary clinical studies reporting patients with TIND. This systematic review was conducted and reported in accordance with the Preferred Reporting Items for Systematic Reviews and Meta-Analyses (PRISMA) 2020 statement [27]. The study protocol was registered retrospectively in the Open Science Framework Registries on 18 August 2026, after completion of the literature search and study selection [28].

The complete PRISMA 2020 checklist is provided in Table S1 in the Supplementary Materials. Eligibility criteria, outcomes and the data-extraction template were agreed among the authors before screening began. PubMed, Scopus, Web of Science and Google Scholar were searched from 1980 to 2026, utilizing a combination of keywords and MeSH terms, such as “treatment-induced neuropathy of diabetes”, “TIND”, “insulin neuritis”, “insulin-induced neuropathy” and “acute painful neuropathy after glycemic correction”. The date of last search was 9 August 2026. Methodological quality was assessed with the tool proposed by Murad et al. for case reports and case series [29], which comprises eight items in four domains: selection, ascertainment, causality and reporting. Overall quality was rated on the three items most relevant to diagnostic certainty in TIND: (1) ascertainment of the exposure (documented HbA1c fall), (2) ascertainment of the outcome (neuropathy) and (3) exclusion of alternative causes. Studies fulfilling all three were rated good, those fulfilling two moderate and the remainder poor; no summary score was calculated. A draft assessment was prepared with the assistance of a large language model (Claude Opus 5.5, Anthropic; accessed October 2026) and was independently verified and corrected by two investigators (N.C.D., R.M.). Agreement was 94% (353/376 items; Cohen’s κ = 0.92), and disagreements were resolved by consensus. Each publication was also classified according to the Gibbons–Freeman criteria [8]. No study was excluded on the basis of quality (Table S3).

The review question was framed using the population–exposure–outcome (PEO) framework, recommended when no intervention or comparator is evaluated [30]. In patients with type 1 or type 2 diabetes (P) who developed TIND after a rapid improvement in glycemic control, documented by a reduction in blood glucose and/or HbA1c following the initiation or intensification of any glucose-lowering treatment (E), what are the demographic characteristics, type of diabetes, comorbidities and microvascular complications, magnitude and rate of HbA1c lowering, time to symptom onset, clinical phenotype, outcome and management (O)? Eligible publications were primary clinical studies of any design (case reports, case series, observational studies and clinical trials), published in English or French, that reported clinical data on patients with TIND. Review articles, commentaries, conference abstracts without full text, studies without clinical outcome data and publications in other languages were excluded. Two observational studies were included: a retrospective cohort [8] and a case–control study of neuropathies associated with glucagon-like peptide-1 receptor agonists (GLP-1RAs) [31]. From the case–control study, only patients with lumbosacral radiculoplexus neuropathy after HbA1c reduction were included, in line with other included reports, and the control group was not used [32,33]. Data were extracted by one investigator into a pre-specified template, and all extracted variables were independently verified by a second investigator. Discrepancies were resolved by consensus. During revision, all entries were re-checked against the full texts, and the resulting corrections were incorporated into Table A1. The extracted data included the author, publication year, patient demographics, diabetes type, clinical presentation, symptom onset time, associated conditions, management modalities, and outcomes. Descriptive statistics were applied to summarize key factors. Where individual patient data were available, variables were reported as ranges. Where only summary statistics data were reported in the source publication, these are reported as published. The type of diabetes was recorded as stated in the source publication. In cases where it was not explicitly specified, the information was noted as not reported and was not inferred based on the treatment. No inferential statistical analyses were conducted due to heterogeneity and the prevalence of case reports and limited case series. To assess the potential effect of overlapping populations reported from the same center [8,22], descriptive totals were recalculated after excluding the cohort reported by Gibbons and Freeman (2010) [22].

The following changes were made relative to the registered protocol [28]: (1) the review question, registered in the population-intervention-comparator-outcome (PICO) format, was reframed using the PEO framework and the objectives were specified, without changes to the eligibility criteria; (2) a sensitivity analysis excluding a potentially overlapping cohort was added [22]; (3) the type of diabetes was recorded as “not reported” when it was not explicitly stated in the source publication; and (4) methodological quality was assessed with the tool of Murad et al. [29], instead of the Cochrane risk-of-bias tool listed in the registration, which is designed for randomized trials.

During the preparation of this work, the authors used Claude (Anthropic; Claude Opus 5.5, accessed August–October 2026) to improve the language and structure of the manuscript, to assist in literature searches and in the verification of extracted data against the source publications, to perform descriptive calculations, to draft Table 2 and Figure 2, and to prepare a draft of the methodological quality assessment, which was independently verified and corrected by two authors. After using this tool, the authors reviewed and edited the content as needed, verified the accuracy and integrity of all output (including all references and any synthesized claims), and take full responsibility for the content of the publication.

## 3. Case Presentation

### 3.1. Case 1

A 63-year-old man presented to the Emergency Department of the Emergency County Hospital, Cluj-Napoca, Romania with syncope and generalized malaise, preceded by several months of polydipsia, polyuria, and cold sensations in his feet. His medical history was notable for a myocardial infarction 13 years earlier, dyslipidemia, obesity and arterial hypertension, with no prior diagnosis of diabetes mellitus.

At admission, blood glucose was 400 mg/dL, with positive blood and urine ketones and glycosuria. HbA1c was markedly elevated, reaching 22.74% (reference range 4–6%) and it was measured using commercially available methods at the Central Laboratory of the Emergency County Hospital Cluj, Romania (Beckman Coulter AU480 Chemistry Analyzer, Brea, CA, USA). Physical examination revealed moderate hepatomegaly, without other abnormalities. A diagnosis of new-onset diabetes mellitus was established. A basal–bolus insulin therapy was initiated, resulting in glycemic improvement over the following days, with blood glucose levels ranging between 100–165 mg/dL. The patient was discharged from the hospital after five days. Due to concerns regarding hypoglycemia, insulin was discontinued one month later, and oral antidiabetic therapy with gliclazide and metformin was started.

Within weeks of initiating glucose-lowering treatment, the patient developed severe bilateral burning pain and paresthesia in the lower limbs, associated with marked allodynia and progressive functional weakness, which continued to worsen over the following months. Neurological examination revealed severe lancinating pain involving both distal and proximal lower limbs, hyperesthesia, reduced patellar reflexes, absent Achilles reflexes, and distal sensory loss across all modalities. Neuropathic pain was assessed using the DN4 questionnaire [24,25], yielding a score of 8/10, and the modified Toronto Clinical Neuropathy Score (mTCNS) was 22, consistent with severe neuropathy [26]. The patient reported significantly reduced quality of life due to neuropathic pain with substantial reduction of daily activities and loss of appetite which led to a 20 kg weight loss over two months. In addition, autonomic symptoms, including constipation and orthostatic dizziness, were reported.

An extensive etiological work-up, including rheumatoid factor, antinuclear antibodies, erythrocyte sedimentation rate, C-reactive protein, creatine kinase, vitamin B12, vitamin B1, and serum protein electrophoresis, yielded normal results. Thyroid-stimulating hormone and thyroid peroxidase antibodies were mildly elevated, with normal free thyroxine and triiodothyronine levels. Subsequent laboratory assessment showed an HbA1c level of 7.44%, reflecting a significant reduction from baseline 22.74% over a two-month period.

NCS showed a slight reduction in motor and sensory nerve conduction velocity (NCV) at baseline and at 1-year intervals (Table 1). These findings indicated a length-dependent axonal sensorimotor polyneuropathy with mild conduction slowing, not meeting electrodiagnostic criteria for demyelination [34]. Clinically, burning pain, allodynia and autonomic symptoms suggested small-fiber involvement, while reduced reflexes and loss of all sensory modalities indicated concomitant large-fiber involvement. Cerebrospinal fluid analysis following lumbar puncture showed no albumin cytological dissociation or other abnormalities. Cervical and thoracic spinal magnetic resonance imaging was unremarkable.

Based on the clinical course, in the context of rapid HbA1c lowering and EDX evolution, a diagnosis of TIND was established. Treatment for neuropathic pain with pregabalin and tramadol was initiated, leading to significant clinical improvement over the subsequent year.

At two-year follow-up, the patient showed marked clinical improvement, with a DN4 score of 4/10 and a mTCNS of 12. Glycemic control remained satisfactory under oral antidiabetic therapy combined with 8 IU of long-acting insulin, with an HbA1c of 6.29%. NCS show a relative improvement at 2 years in distal compound muscle action potential (CMAP) amplitude, compared to the initial EDX assessment (Table 1).

### 3.2. Case 2

We present the case of a 43-year-old man who was admitted to the Emergency Department of the Emergency Clinical County Hospital, Cluj-Napoca, Romania with generalized malaise and a plasma glucose of 487 mg/dL, for which rapid-acting insulin was administered. His HbA1c at presentation was 13% (reference range 4–6%). The patient had no significant medical history. Family history was notable for T2DM in both parents, managed with oral antihyperglycemic agents. The patient was diagnosed with T2DM and initiated oral antihyperglycemic therapy with gliclazide and metformin, resulting in significant improvement in glycemic control.

Six months after diagnosis, the patient developed severe throbbing pain, paresthesia, and a “cardboard-like” sensation, suggestive of dysesthetic sensory disturbance in the feet and lower legs, progressively worsening over the preceding month. Additionally, he reported marked fatigue, anorexia, and a 15 kg weight loss over several months.

On general examination, blood pressure was 122/83 mmHg. Neurological assessment revealed normal muscle strength, decreased Achilles reflex on the right and absent on the left, and reduced sensation to light touch and pinprick in the feet and distal calves. Vibration sense was impaired bilaterally (5/8 at the hallux, 7/8 at the knees). Cranial nerves and cerebellar function were intact. The patient reported dizziness upon standing, suggestive of orthostatic hypotension, as well as hyperhidrosis of the hands and feet and episodic diarrhea alternating with constipation. Neuropathic pain assessment using the DN4 yielded a score of 6/10, and the mTCNS was 21, consistent with severe neuropathy.

Laboratory evaluation on the second admission demonstrated fasting glucose levels between 108 and 160 mg/dL. HbA1c at that time was 6.61%, representing a reduction of 6.4 percentage points over a six-month period. An extended serology work-up was performed in order to exclude other potential causes. Serology was positive for hepatitis C virus (HCV) with antibody titer at 37 IU (reference range < 0.9), with no evidence of cryoglobulinemia; HCV viral load was not measured. Vitamin B12 level was moderately decreased, with a measured level of 131 pg/mL (reference range 200–900 pg/mL), prompting initiation of vitamin B12 supplementation. Homocysteine or methylmalonic acid levels were not measured.

NCS demonstrated reduced distal CMAP and sensory nerve action potential (SNAP) amplitudes in the nerves examined, associated with mild slowing of motor and sensory NCV in the lower limbs, remaining within the range attributable to loss of large-diameter axons (Table 1). These findings were consistent with an length-dependent axonal sensorimotor polyneuropathy exhibiting mild slowing of conduction velocity, not meeting criteria for demyelination [34]. Lumbar puncture was proposed as part of the diagnostic evaluation but was declined by the patient. Moreover, formal autonomic testing, including sympathetic skin response and sudomotor function assessment (e.g., electrochemical skin conductance testing), was not available at our center at that time.

Oral antidiabetic therapy was maintained. In addition to vitamin B12 supplementation, treatment with alpha-lipoic acid was initiated. Pregabalin was started during hospitalization and continued after discharge for symptomatic management of neuropathic pain.

At the two-year follow-up, the patient reported near-complete resolution of clinical symptoms. The DN4 score had improved to 2/10, with resolution of neuropathic pain, and the TCNS had decreased to 3, below the threshold for neuropathy. NCS showed improvement in upper-limb distal CMAP amplitude with continued decline in distal sensory amplitude. HCV seropositivity, without measurement of viral load, and vitamin B12 deficiency may have contributed to the neuropathy; nevertheless, its acute onset during rapid glycemic correction made TIND the most likely diagnosis. 

## 4. Results of the Systematic Review

### Population

**Figure 1:**
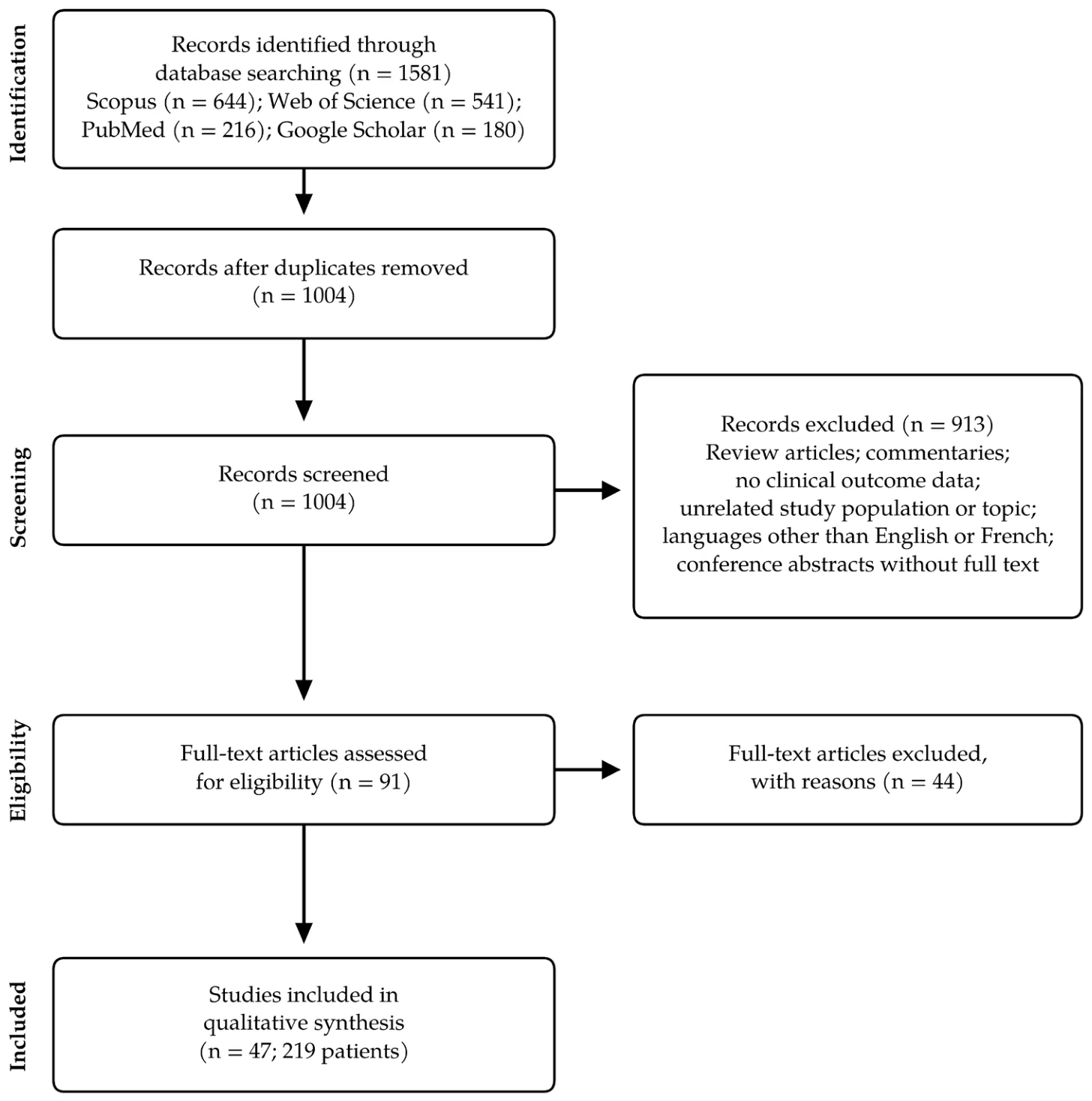
PRISMA flow diagram of the study selection process.

A PRISMA flowchart illustrating the study selection process is presented in Figure 1.

The initial search yielded 1581 records from four electronic databases: 644 records from Scopus, 541 records from Web of Science, 216 records from PubMed and 180 records from Google Scholar. After removal of duplicates, 1004 records remained for title and abstract screening. Following screening, 913 records were excluded due to various reasons, including review articles, commentaries, absence of clinical outcomes, irrelevant study population or topic, publication in languages other than English or French, and conference abstracts without an available full text. A total of 91 articles were assessed for eligibility, of which 44 were excluded for different reasons. Ultimately, 47 studies were included in the qualitative synthesis. Study selection was performed independently by two investigators, with discrepancies resolved by consensus.

A total of 47 publications were included (33 case reports, 12 case series, one retrospective cohort and one case–control study), comprising 219 patients with TIND. Two cohorts of patients were reported by the same author from the same center [8,22]. Since the 2015 report included all patients evaluated between 2008–2012, and the 2010 report did not specify the inclusion dates, any potential overlap could neither be confirmed nor excluded. Therefore, in a sensitivity analysis that excluded the 16 patients mentioned in [22], the series comprised a total of 203 patients (58.6% women; 60.1% type 1 diabetes, 36.5% type 2 diabetes, 3.4% type unspecified), with proportions differing by less than 1% from those in the full series. The principal characteristics of the included publications are summarized in Table 2, and full case-level data are provided in Appendix A (Table A1).

Among reports providing individual patients’ ages, ages at symptoms onset ranged from 9 to 72 years. Females accounted for 58% of the patients included in our analysis. T1DM was present in 131 (59.8%) patients and T2DM in 81 (37.0%); the type of diabetes was not reported in 7 (3.2%) patients. Data regarding duration of diabetes at symptoms onset was heterogeneous. Among available data, duration varied widely, ranging from several weeks from diagnosis to more than 20 years. Also, these proportions describe published cases, which are subject to selection and reporting bias, and should not be interpreted as estimates of the prevalence or characteristics of TIND in clinical populations.

Painful neuropathy with autonomic dysfunction was the most frequent phenotype (14 publications, 151 patients), followed by isolated painful neuropathy (12 publications, 19 patients). Additional motor manifestations, specific characteristics, or other features were described in 13 publications; focal or proximal presentations were reported in 6 publications; and the absence of pain was noted in 2 (Table 2). Across the 31 case presentations, the median decrease in HbA1c levels was 7.3 percentage points (range 2.2–12.5), and the onset of symptoms was reported within an 8-week interval in 26 of the 47 publications. The most frequently used treatments were gabapentinoids (28 publications), antidepressants (26), and opioids (23).

Methodological quality was rated good in 14 of 47 publications, moderate in 21 and poor in 11, mainly because of incomplete exclusion of alternative causes. One publication available only as an abstract could not be assessed (Table S3). Moreover, when the Gibbons–Freeman criteria [8] were applied, 25 publications described cases fulfilling all three criteria, 8 met the HbA1c criterion but did not report the onset interval precisely, 3 reported onset beyond the 8-week window, and 8 papers provided insufficient data for assessment. In 3 case series, the criteria were fulfilled by only some patients (Table S3).

## 5. Discussion

This analysis identified 47 publications describing 219 patients who developed painful neuropathy and/or autonomic neuropathy following a rapid improvement in glycemic control. Since nearly all these data originate from case reports and small patient series, they document a temporal association but cannot establish its strength or a robust causal relationship. The frequency of TIND is uncertain: 10.9% of patients referred to a tertiary-care clinic specializing in diabetic neuropathy met the diagnostic criteria [8]. We believe this figure is likely an overestimate and cannot be extrapolated to the entire population of patients with diabetes due to selection bias, as patients are referred to specialized centers because they present with complex forms of the disease. Furthermore, among the published cases, type 1 diabetes predominated (59.8%). Moreover, it was primarily observed in young patients (median age of 28 years in the case reports), with a diabetes duration ranging from onset to over 20 years (Table 2). In contrast, both patients in our study were middle-aged men with recently diagnosed type 2 diabetes. This discrepancy may reflect reporting patterns, as it is possible that TIND is underdiagnosed in older adults with type 2 diabetes, specifically when neuropathic symptoms are erroneously attributed to chronic diabetic polyneuropathy. Specific contexts included the following: eating disorders [22,59], restrictive diets [45], weight loss associated with the use of GLP-1 receptor agonists or bariatric surgery [31,67], as well as pancreatic transplantation [60,69]. Microvascular complications were frequent in the larger cohorts [8,22], but TIND also occurred in newly diagnosed patients without such complications [43,46,51], as in our two patients, who had no retinopathy or nephropathy at diagnosis.

The glycemic change preceding TIND was substantial. Thus, among case reports, the median HbA1c fall was 7.3% usually within 3 months (Table 2). Moreover, in the largest cohort, the absolute risk of TIND increased from approximately 20% with a fall of 2–3 points over 3 months to more than 80% with a fall of ≥4 points [8], suggesting a dose–response relationship. Case 1 had the largest fall reported to date (15.3% over 2 months, versus a maximum of 12.5 in published cases [33]) and developed symptoms within weeks, fulfilling all three Gibbons–Freeman criteria [8]. In Case 2, the fall of 6.4 points over 6 months met the HbA1c criterion [8,15]. However, as HbA1c was measured only at diagnosis and at 6 months, the interval between the fall and symptom onset could not be established and this case is best regarded as compatible with an expanded TIND phenotype. Timing also varied in the literature: onset was reported within 8 weeks in 26 publications, but after several months in seven reports [31,38,49,50,55,65,72] and within days in four [39,45,60,61]. Although painful neuropathy with autonomic dysfunction was the predominant phenotype, published cases also described motor weakness [61,62,64,65,67,68], neuropathic cachexia [58,59] and focal or proximal presentations, including lumbosacral radiculoplexus and truncal neuropathies [31,32,69,70,71], while two patients had no neuropathic pain [73,74]. In our study, both patients presented with the predominant phenotype, painful length-dependent neuropathy with orthostatic and gastrointestinal symptoms. Additionally, Case 1 developed functional weakness and a 20 kg weight loss. Moreover, according to the studies, TIND is primarily a small-fiber and autonomic neuropathy [8]. In our patients, small-fiber and autonomic involvement was clinically inferred, while serial NCS showed a length-dependent axonal sensorimotor polyneuropathy with mild conduction slowing. Furthermore, large-fiber abnormalities have been reported in a subset of patients with TIND [8,22] and may reflect preexisting diabetic polyneuropathy unmasked or aggravated by rapid glycemic correction [75,76]. In our cases at 2 years, CMAP amplitudes improved in the median and peroneal nerves in Case 1 and in the upper limbs in Case 2, while sural SNAP responses were absent in both patients and tibial CMAP amplitudes declined in Case 2 (Table 1), indicating incomplete recovery of large-fiber involvement.

The mechanisms presented in the Introduction, have been refined by indirect evidence. Thus, recent reviews describe a neurovascular model in which arteriovenous shunting, leaky epineurial neovessels and ischemia–reperfusion injury during abrupt glycemic normalization reduce endoneurial oxygen delivery [11,21]. This model is consistent with the concurrent microvascular deterioration reported in the included studies. Therefore, worsening retinopathy was described in 10 publications [8,12,22,36,46,47,51,55,61,64] and albuminuria or proteinuria in four [8,46,55,64]. At the same time, in the largest cohort, retinopathy increased from 39/104 to 94/104 patients and microalbuminuria from 18/104 to 87/104 within 12 months of TIND onset [8]. This is analogous to the early worsening of retinopathy and nephropathy described following rapid correction of blood glucose levels [21,77]. Oxidative stress and inflammation, central to chronic diabetic neuropathy may also contribute. In rats, rapid HbA1c reduction induced macrophage infiltration of the sciatic nerve [5,14]. In addition, insulin enhanced acid-sensing ion channel currents and nociceptive behavior in rat sensory neurons, and circulating pro-inflammatory cytokines increased with the severity of cardiovascular autonomic neuropathy in T2DM [78,79]. However, none of these data derive from patients with TIND. EDX findings are also variable, ranging from normal nerve conduction in early, predominantly small-fiber TIND to axonal sensorimotor changes with mild conduction slowing [21,80]. Finally, micronutrient deficiencies may lower the threshold for nerve injury or confound the diagnosis. Thus, iron and vitamin D deficiencies were reported in isolated TIND cases, nutritional deficiencies accompanied GLP-1 receptor agonist-related weight loss, and vitamin B12 deficiency, present in our second patient, has been associated with the onset and worsening of diabetic neuropathy [65,67,72,81].

In our patients, alternative diagnoses were considered. In Case 1, progressive weakness and reduced reflexes raised the possibility of chronic inflammatory demyelinating polyneuropathy (CIDP), which typically progresses over more than 8 weeks and is supported by demyelinating EDX features and albuminocytological dissociation [34]. At the same time, diabetes may coexist with or mimic CIDP, and TIND–CIDP overlap has been reported [66,82]. However, in our case, serial NCS showed no demyelinating features and cerebrospinal fluid was normal, with gradual improvement of symptoms. In Case 2, two potential, overlapping causes were identified. HCV-related neuropathy usually progresses slowly and when it is acute, it is typically vasculitic and associated with cryoglobulinemia, which was excluded [83,84]. On the other hand, vitamin B12 deficiency usually causes a slowly progressive, predominantly large-fiber sensory neuropathy [81,85]. The acute onset during marked HbA1c reduction and the prominent autonomic symptoms favor TIND, but a contribution of both factors cannot be excluded.

Management in the published cases was symptomatic and heterogeneous (Table 2). Thus, gabapentinoids were the most frequently used agents, followed by antidepressants and opioids, usually in combination, reflecting the poor responsiveness of pain in TIND [8]. In contrast, both our patients were treated with pregabalin, combined with tramadol, and neither received an antidepressant. Pregabalin was associated with marked pain relief in two reports [42,50] but no comparative data support the superiority of any agent in TIND, and treatment follows recommendations for painful diabetic neuropathy [86]. Autonomic-directed therapy was reported in five publications [49,56,61,68,73] and deliberate relaxation of glycemic targets in four [31,38,51,74], although there is no evidence that relaxation improves outcomes [21]. Moreover, in randomized trials in diabetic polyneuropathy, alpha-lipoic acid, an antioxidant given to our second patient, improved neuropathic symptoms but not NCV [87]. However, it has not been studied in TIND, so outcomes studies should be interpreted cautiously. Thus, among case reports with documented outcomes, recovery was complete in 8 of 32 patients, partial in 21 and absent in 3. Outcome data were lacking for 136 of 219 patients overall. In cohort data, pain and autonomic symptoms improved substantially over 18 months and long-term improvement depended on stable glycemic control [9,22]. Both our patients improved substantially at 2 years without relaxation of glycemic targets.

**Figure 2:**
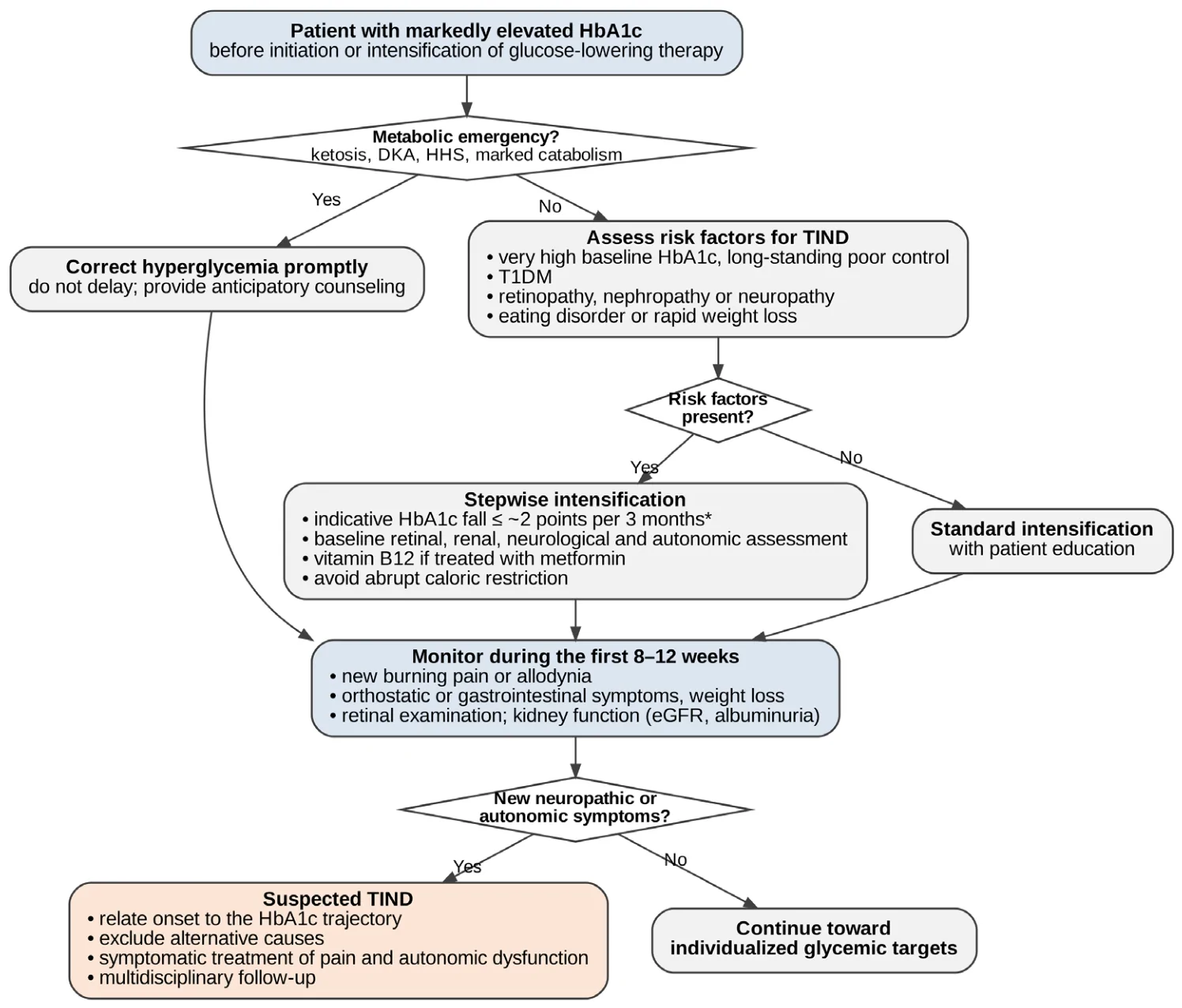
Proposed risk-adapted approach to glycemic correction in patients with markedly elevated HbA1c, aimed at the prevention and early recognition of treatment-induced neuropathy of diabetes (TIND). The approach is based on expert opinion and on the descriptive data of this review and has not been validated [8,21,22,31,45,67,81]. * Indicative threshold; urgent correction should not be delayed when clinically required. DKA, diabetic ketoacidosis; eGFR, estimated glomerular filtration rate; HbA1c, glycated hemoglobin; HHS, hyperosmolar hyperglycemic state; T1DM, type 1 diabetes mellitus.

The most consistent finding of this review is the temporal association between TIND and a rapid fall in HbA1c, which supports a cautious, risk-adapted approach to glycemic correction (Figure 2). At present, no safe rate of HbA1c reduction has been established, but in the largest cohort, a fall of 2–3 percentage points over 3 months carried a TIND risk of approximately 20% [8]. Moreover, expert opinion suggests avoiding falls greater than 2 points over 3 months in high-risk patients without a metabolic emergency [21]. At the same time, higher risk is associated with very high baseline HbA1c, long-standing poor control, T1DM, microvascular complications, eating disorders and rapid weight loss [8,21]. When urgent correction is needed, as in our first patient with ketosis, it should not be delayed. However, it must be accompanied by prior counseling and monitoring over the first 8–12 weeks, specifically checking for the onset of neuropathic pain, orthostatic and gastrointestinal symptoms, retinopathy, and changes in renal function [21]. Documented deficiencies, such as vitamin B12 deficiency in metformin-treated patients, should be corrected and abrupt caloric restriction avoided [31,45,67]. Currently, there is no evidence to suggest that antioxidants or other dietary supplements can prevent TIND, though these options maybe considered at some point.

Our case series has several limitations. Small-fiber and autonomic involvement was established on clinical grounds only, as skin biopsy and quantitative sudomotor and cardiovascular autonomic testing were not available [17,20]. In Case 2, the interval between the HbA1c fall and symptom onset could not be determined, and HCV seropositivity, vitamin B12 deficiency and concomitant supplementation may have contributed to the neuropathy and its improvement. Thus, the diagnosis of TIND is less certain than in Case 1.

The systematic review also has limitations. The search was restricted to publications in English and French. Methodological quality was moderate or poor in 32 of 47 publications, mainly because alternative causes were incompletely excluded or the HbA1c fall was not fully documented. Data from the included studies demonstrated significant heterogeneity, mostly due to non-standardized symptom definitions and inconsistent reporting of follow-up evolution. Most studies did not use validated pain assessment scales, limiting quantitative comparison and meta-analysis. Additionally, the evidence derives mainly from case reports and small series and is prone to publication bias: severe, unusual or successfully diagnosed cases are more likely to be published than mild or unrecognized ones. This can lead to an overrepresentation of severe and atypical phenotypes and may distort the prognosis in either direction. The aggregate proportions should be interpreted as descriptive summaries of published cases rather than as unbiased estimates of the frequency and characteristics of TIND. The two cohorts reported by Gibbons and Freeman originate from the same tertiary center and may overlap [8,22]. Moreover, the degree of this overlap could not be determined, although a sensitivity analysis excluding the smaller cohort did not substantially change the descriptive proportions. Follow-up data were not reported for 136 of the 219 patients, including those in the two largest cohorts [8,31]. Consequently, the figures regarding recovery reflect the literature based on case reports, rather than the population as a whole. Moreover, the Gibbons–Freeman criteria were derived from a single tertiary cohort [8]. Several included cases did not strictly meet them, because symptoms began after several months [31,38,49,50,55,65,72] or the HbA1c change was not quantified [11,35,37,40,74], and some patients had coexisting neuropathic conditions, such as CIDP [66], diabetic amyotrophy [62,64] or pre-existing diabetic polyneuropathy [53,56,65,72]. Therefore, diagnostic misclassification cannot be excluded. Prospective studies with standardized assessment of pain, autonomic function and glycemic trajectory are needed to define safe rates of glycemic correction and to identify patients at risk.

## 6. Conclusions

TIND is an under-recognized iatrogenic neuropathy that occurs in both T1DM and T2DM after rapid glycemic correction. In the published cases and in our two patients, painful and frequently autonomic neuropathy followed rapid reduction in HbA1c, most often within weeks, As the evidence derives almost entirely from case reports and small series, it documents a recurrent temporal association but does not establish causality, incidence or prognosis. Moreover, improvement was frequently reported, but recovery was often incomplete and outcome data were missing for most patients. TIND should be considered in patients who develop acute neuropathic pain or autonomic symptoms after a rapid fall in HbA1c. Likewise, monitored glycemic correction may be reasonable in high-risk patients when no metabolic emergency is present.

## Figures and Tables

**Table 1:** Nerve conduction studies of the two patients at baseline and during follow-up.

	Case 1			Case 2	
Nerve	Baseline	1 Year	2 Years	Baseline	2 Years
***Motor NCS: distal CMAP amplitude (mV)/NCV (m/s)***					
Median R	4.3/45	4.6/43	6.1/47	3.5/38	4.7/45
Median L	4.5/44	4.4/44	6.5/50	3.0/40	3.9/50
Ulnar R	8.8/50	9.4/47	9.1/48	9.1/43	12.1/51
Ulnar L	11.0/53	10.2/54	11.2/55	8.4/49	10.3/54
Peroneal R	2.7/37	2.4/34	3.2/38	3.7/32	4.5/38
Peroneal L	1.7/35	1.7/34	2.7/38	5.3/32	4.1/34
Tibial R	4.4/37	3.2/34	3.5/40	1.4/35	1.2/30
Tibial L	4.9/35	4.9/36	5.9/36	1.0/36	0.3/30
***Sensory NCS: SNAP amplitude (µV)/NCV (m/s)***					
Median R	3/38	2/32	2/37	11/46	11/45
Median L	3/37	3/40	2/37	9/42	9/50
Ulnar R	3/40	2/41	2/44	9/43	6/40
Ulnar L	3/39	2/41	NR	10/43	9/44
Sural R	3/41	3/45	NR	3/30	NR
Sural L	NR	3/36	NR	NR	NR

Values are given as amplitude and conduction velocity. Laboratory lower limits of normal: distal CMAP amplitude: median 4 mV, ulnar 6 mV, peroneal 3 mV and tibial 6 mV; SNAP amplitude: median, ulnar and sural 10 µV; motor and sensory conduction velocity: 45 m/s in the upper limbs and 40 m/s in the lower limbs. CMAP, compound muscle action potential; L, left; NCS, nerve conduction study; NCV, nerve conduction velocity; NR, no response; R, right; SNAP, sensory nerve action potential.

**Table 2:** Principal characteristics of the published cases of treatment-induced neuropathy of diabetes, grouped by clinical phenotype, and of the two present cases.

Study [Ref.]	Participants: N/Sex; Age (Years); Diabetes Type; Diabetes Duration (Years)	HbA1c Fall (Percentage Points)/Interval; Symptom Onset	Additional Features and Relevant Context	Treatment	Outcome (Follow-Up)
***Painful neuropathy (12 publications, 19 patients)***					
Dabby et al., 2009 [35]	6; 5M/1F; 27–58; DM type NR ^†^; 3–5	NR (rapid correction); 2–4 wk	–	GBP, PGB, TCA, SNRI, OP, CBZ	Resolution over 3–8 mo
Mudenha & Mansell, 2016 [36]	2; 2F; 28 and 30; T1DM; 3 and 23	7.2/3 mo; 4.6/7 wk; 2–3 wk	Pregnancy (2/2)	GBP, TCA, OP	Complete at 10 wk (1); partial at 6 mo (1)
Van Haren, 2017 [37]	2; 2F; 15 and 22; T1DM 2; DM NR	NR (rapid correction); Several weeks	–	GBP, PGB, TCA, SNRI, OP, other	Improvement over several months
Llewelyn et al., 1986 [12]	18 F; T1DM; 8	9.5 (HbA1)/6 wk; Several weeks	–	OP, CBZ, other	Improvement at 6 mo
Wilson et al., 2003 [38]	14 M; T1DM; 1	6.5/9 wk; 3 mo	–	GBP, OP, GR, other	Improvement at 3 mo
Song et al., 2009 [39]	30 M; T1DM; 15	>2.2/1 mo; 1 wk	–	TCA	Improvement at 1 mo
Rothacker & Kaye, 2014 [40]	20 M; T1DM; 5	NR (insulin pump); Several weeks	Insulin oedema	GBP, SNRI, OP	Improvement at 8 mo
Hernández et al., 2018 [41]	25 M; T1DM; ~3	6.2/2 mo; 2 wk	–	SNRI, OP	Near-complete at 6 mo
Chandler et al., 2020 [42]	18 M; T1DM; new onset	7.3/7 wk; 2 wk	–	GBP, PGB, TCA, OP, CBZ	Gradual improvement over 18 mo
Ferreira et al., 2021 [43]	32 M; T1DM; 3	4.8/6 mo; 1 mo	–	TCA	Complete at 6 mo
Abdelhamid et al., 2023 [44]	60s M; T2DM ^‡^; <1	>7.7/4 mo; 6 wk	–	PGB, SNRI, OP	Near-complete at 1 y
Sahyouni et al., 2024 [45]	50 M; T2DM; new onset	8.0/3 mo; Several days	Low-carbohydrate diet	GBP, other	Partial at 1 y
***Painful neuropathy with autonomic dysfunction (14 publications, 151 patients)***					
Gibbons & Freeman, 2015 [8]	104; 31M/73F; mean age 25 (T1DM) and 51 (T2DM); T1DM 76, T2DM 28; DM 9.6 and 5.4 (means)	≥2/≤3 mo (inclusion criterion); ≤8 wk	Retinopathy, microalbuminuria frequent	OP, anticonvulsants, antidepressants	NR (no follow-up reported)
Gibbons & Freeman, 2010 [22]	16; 7M/9F; 19–58 (mean 35); T1DM 9, T2DM 7; DM 3–13	≥2/≤3 mo (inclusion criterion); 6–8 wk	Eating disorder 7/16; retinopathy, microalbuminuria	OP, anticonvulsants, antidepressants	Pain improved by ~15 mo, autonomic symptoms by ~18 mo
Alexandrou et al., 2020 [46]	7; 5M/2F; 9–22; T1DM 7; DM 1 mo–5	>2/3 mo; 3–8 wk	–	GBP, PGB, SNRI	Resolution in 6/7 over 3–12 mo; 1 unresolved
Tesfaye et al., 1996 [11]	5; 3M/2F; 34–64; T1DM 2, T2DM 3; DM 0–5	NR (rapid insulin titration); 4–8 wk	–	TCA, OP	Marked improvement over 6–9 mo
Koh et al., 2019 [47]	5; 3M/2F; 43–72; T2DM 5; DM 6–20	4.7 (median)/1–3 mo; 2–8 wk	Nosocomial, during acute illness	NR	NR
Tran et al., 2015 [48]	4; 3M/1F; 41–57; T1DM 2, T2DM 2; DM 1 mo–5	7.2 (mean)/4.5 mo (mean); 4–6 wk	–	GBP, PGB, SNRI, OP	Variable recovery at 12–14 mo
Hwang & Davies, 2016 [49]	2; 2F; 22 and 24; T1DM 2; DM 16	>2/12 mo; Several months	–	GBP, PGB, AUT	Improvement after 1–9 mo
Aladdin et al., 2017 [50]	2; 1M/1F; 43 and 46; T1DM 1, NR 1; DM 14–16	8.2/3 mo; NR; Several weeks; 5 mo	–	GBP, PGB, OP	Complete at 2 y (1); partial at 1 y (1)
Varadharaju et al., 2019 [51]	16 F; T1DM; new onset	10.4/2 mo; 5 wk	–	GBP, PGB, TCA, GR	Complete at 2 mo
Guisado Vasco et al., 2020 [52]	23 F; T1DM; 5	3.0/<2 mo; Few weeks	–	GBP, OP	Improvement at 6 mo
Oleynick et al., 2020 [53]	51 F; T1DM; 15	5.1/6 mo; 2 wk	Worsening of pre-existing neuropathy; dysphagia	GBP, PGB, SNRI, OP	Return to baseline
Kim et al., 2020 [54]	24 F; T1DM; 10	9.4/3 mo; 1 mo	–	PGB, TCA, OP	Complete at 1 mo
Stainforth-Dubois & McDonald, 2021 [55]	20 F; T1DM; new onset	10.3/3 mo; 3–6 mo	–	SNRI	Improvement at 18 mo
MacIsaac et al., 2026 [56]	51 M; T2DM; NR	9.4/3 mo; 3 wk	Autonomic-predominant	PGB, AUT	Partial at 6 mo
***Painful neuropathy with motor, cachectic or other additional features (±autonomic dysfunction) (13 publications, 14 patients)***					
Yip et al., 2011 [57]	2; 2F; 18 and 27; T1DM 2; DM 0 and ~11	NR; 7.4/NR; 1 mo	Pseudo-phaeochromocytoma; severe weight loss; Graves disease (1)	GBP, TCA, OP	Complete within 1 y
Yuen et al., 2001 [58]	32 M; T1DM; new onset	7.8/6 wk; 6 wk	Myalgia, neuropathic cachexia; autonomic dysfunction; cataracts	symptomatic NOS	Improvement at 6 mo
Deorchis et al., 2013 [59]	16 F; T1DM; 2	7.2/NR; Subacute	Neuropathic cachexia (weight loss)	PGB, TCA, other	Partial at 6 mo
Meillet et al., 2019 [60]	35 F; T1DM; 23	9.9/2 mo; 10 d	Ataxic gait; autonomic dysfunction; islet transplantation	PGB, TCA, SNRI, OP	No recovery at 6 y
Triplett & Yiannikas, 2019 [61]	27 F; T1DM; 20	7.6/6 wk; Several days	Multifocal weakness; autonomic dysfunction; after DKA	AUT	No recovery at 8 wk; lost to follow-up
Shimizu et al., 2019 [62]	28 F; T1DM; 18	7.0/2 mo; 2 mo	Lower-limb weakness (diabetic amyotrophy); pontine lesion	other	Partial at 1 mo
Rasmussen et al., 2022 [63]	16 F; T1DM; new onset	9.8/3 mo; 1 mo	Restless legs syndrome; weight loss; autonomic dysfunction	GBP, PGB, TCA	Partial at 18 mo
Pereira et al., 2023 [64]	40 F; T2DM; 12	6.1/1 mo; Rapid	Asymmetric proximal weakness (diabetic amyotrophy); autonomic dysfunction; pregnancy	symptomatic NOS	Partial at 10 wk
Schueller et al., 2025 [65]	50 F; T2DM; DM NR	7.9/NR; 5 mo	Lower-limb weakness; orthostatic hypotension	GBP, SNRI	Some improvement
Hu et al., 2025 [66]	26 M; T1DM; 3	2.7/2 mo; 2 mo	Perineal pain; subsequent CIDP	GBP, PGB, OP, CBZ, CS, other	Improvement at 5 mo
Zahir et al., 2025 [67]	37 F; T2DM; NR	7.0/10 wk; Several weeks	Asymmetric proximal weakness; autonomic dysfunction; GLP-1RA-related weight loss	OP	No recovery at 6 mo
Abohalima et al., 2025 [68]	17 M; T1DM; new onset	5.1/3 mo; 1 mo	Distal lower-limb weakness; abdominal pain; autonomic dysfunction	GBP, TCA, OP, IVIG, AUT	NR
Teixeira et al., 2025 [33]	59 M; T2DM; 20	12.5/3 mo; 2 wk	Features of lumbosacral radiculoplexus neuropathy (proximal thigh pain, atrophy); weight loss	symptomatic NOS	Improvement at 1 y
***Focal, proximal or truncal phenotypes (6 publications, 33 patients)***					
Triplett et al., 2025 [31]	26 (27 episodes); 14M/12F; age 62.6 *; T1DM 1, T2DM 25; DM NR	2.4 (1–8.5)/NR; 6 mo (3–35)	Lumbosacral radiculoplexus neuropathy; GLP-1RA treatment	GR	NR
Siddique et al., 2020 [32]	3; 3M; 56–67; T2DM 3; DM 8 mo–2	>2/3 mo; Several weeks	Painful neuropathy (1), LRPN (1), oculomotor palsy (1)	PGB	Improvement over 3–12 mo
Ndip et al., 2009 [69]	31 F; T1DM; 28	4.9/3 mo; NR	Thoracic polyradiculoneuropathy; pancreas transplantation	PGB, TCA	Complete within weeks
Aslam et al., 2014 [70]	31 M; T1DM; new onset	7.3/2 mo; 2 mo	Truncal (abdominal) pain; weight loss	TCA	Partial at 1 mo
Kanbour et al., 2023 [71]	36 M; T1DM; new onset	8.4/1 mo; Several weeks	Painful radiculopathy; restless legs syndrome	GBP, TCA, SNRI, OP, other	Partial at 1 y
Lee et al., 2025 [72]	25 M; T1DM; 16	4.1/10 mo; 8.0/7 mo (2 episodes); Several months	Episode 1 painful neuropathy; episode 2 painful radiculopathy	GBP, PGB, TCA, SNRI, other	Recovery (episode 1); partial at 1 y (episode 2)
***Without neuropathic pain (2 publications, 2 patients)***					
Partridge & Kerr, 2009 [73]	19 F; T1DM; 4	5.8/6 mo; NR	Gastrointestinal autonomic dysfunction; weight loss	AUT	Complete
Broadhead & Devenport, 2021 [74]	66 F; T2DM; 9	NR (baseline > 14)/3 mo; NR	Sensorimotor polyneuropathy	GR	Improvement at 3 wk
***Present cases***					
**Case 1 (present report)**	63 M; T2DM; new onset	15.3/2 mo; Within weeks	Progressive functional weakness; 20 kg weight loss; autonomic dysfunction	PGB, OP	Marked improvement at 2 years (mTCNS 22 → 12)
**Case 2 (present report)**	43 M; T2DM; new onset	6.4/6 mo; ~6 mo after diagnosis	15 kg weight loss; autonomic dysfunction; HCV seropositivity, vitamin B12 deficiency	PGB, ALA, vitamin B12	Near-complete resolution at 2 years (mTCNS 21 → 3)

Studies are grouped by predominant clinical phenotype; within groups, case series precede case reports. HbA1c falls are given in percentage points (NGSP units) and onset as reported in the source publication. Treatment lists agents administered for TIND-related symptoms. Full case-level data, including comorbidities, are provided in Appendix A (Table A1). * As reported in the source publication. ^†^ Diabetes type not stated (insulin in 3, oral agents in 2, diet in 1). ^‡^ Latent autoimmune diabetes in adults, classified as T2DM in the source. HbA1, total glycated hemoglobin. ALA, alpha-lipoic acid; AUT, autonomic-directed therapy; CBZ, carbamazepine; CIDP, chronic inflammatory demyelinating polyradiculoneuropathy; CS, corticosteroids; DKA, diabetic ketoacidosis; DM, diabetes mellitus; GBP, gabapentin; GLP-1RA, glucagon-like peptide-1 receptor agonist; GR, relaxation of glycemic targets; HCV, hepatitis C virus; IVIG, intravenous immunoglobulin; LRPN, lumbosacral radiculoplexus neuropathy; mo, months; NOS, not otherwise specified; NR, not reported; OP, opioids; PGB, pregabalin; SNRI, serotonin–norepinephrine reuptake inhibitor; T1DM/T2DM, type 1/type 2 diabetes mellitus; TCA, tricyclic antidepressant; wk, weeks; y, years; M, male; F, female.

## Data Availability

The data supporting the findings of this study are available from the corresponding authors upon reasonable request. Data are not publicly available due to privacy restrictions applying to individual patient records.

## Supplementary Materials

The following supporting information can be downloaded at: https://www.mdpi.com/article/10.3390/jcm15218760/s1, Table S1: PRISMA 2020 checklist; Table S2: Full electronic search strategy, by database; Table S3: Methodological quality of the included publications, assessed with the tool of Murad et al., and classification according to the Gibbons–Freeman diagnostic criteria.

## Abbreviations

The following abbreviations are used in this manuscript:

CIDP	Chronic inflammatory demyelinating polyradiculoneuropathy
CMAP	Compound muscle action potential
EDX	Electrodiagnosis
DN4	Douleur neuropathique en 4 questions
DKA	Diabetic ketoacidosis
eGFR	Estimated glomerular filtration rate
GLP-1	Glucagon-like peptide-1
HbA1c	Glycated hemoglobin
HCV	Hepatitis C virus
HHS	Hyperosmolar hyperglycemic state
mTCNS	modified Toronto clinical neuropathy score
NCS	Nerve conduction study
NCV	Nerve conduction velocity
PEO	Population exposure outcome
PICO	Population, intervention, comparator, outcome
PRISMA	Preferred reporting items for systematic reviews and meta-analyses
SNAP	Sensory nerve action potential
TIND	Treatment-induced neuropathy of diabetes
T1DM	Type 1 diabetes mellitus
T2DM	Type 2 diabetes mellitus

## Appendix A

**Table A1:** Case-level data of the case reports, case series and case–control study included in the systematic review.

Author (Year)	Study Type	N (pts)	Age (yrs)	Sex		Type of Diabetes			DM Duration	Clinical Presentation	Relevant Comorbidities and Clinical Context Prior to TIND	HbA1c Drop/ Rapid Glycemic Change	Timing of Symptom Onset	Management/Intervention	Outcome/Follow-Up
				M	F	T1 DM	T2 DM	NR *							
Gibbons & Freeman, 2015 [8]	Retrospective cohort	104	25 (mean T1DM) 51 (mean T2DM)	31	73	76	28	0	9.6 (mean T1DM) 5.4 (mean T2DM)	Painful neuropathy Autonomic dysfunction	N-P retinopathy-35/104 P retinopathy-4/104 Microalbuminuria-18/104 HTA-24/28 Hyperlipidemia-25/28	≥2% drop over 3 months	2–8 weeks	Symptomatic treatment, glucose monitoring	NR
Triplett, 2025 [31]	Retrospective case–control	26	62.6 (mean)	14	12	1	25	0	NR	Painful lumbosacral radiculoplexus neuropathy	Retinopathy-2/27 Nephropathy-4/27 D neuropathy-2/27 Gastric by-pass-1/27	2.4%	6 months (3–35 months)	Reduction of antidiabetic medication	NR
Gibbons, 2010 [22]	Case series	16	19–58 35 (mean)	7	9	9	7	0	3–13yrs 7 (mean)	Painful neuropathy Autonomic dysfunction	Diabetic anorexia-7/16 N-P retinopathy-7/16 Microalbuminuria-8/16	≥2% drop over 3 months	6–8 weeks	Symptomatic treatment, glucose monitoring	Pain improvement 15 months Autonomic symptoms 18 months
Alexandrou, 2020 [46]	Case series	7	9–22 17 (mean)	5	2	7	0	0	1 month–5 yrs 1.7 (1.65) (mean)	Painful neuropathy Autonomic dysfunction	N-P retinopathy-2/7 Macular edema-1/7 Cataract-1/7 Microalbuminuria-3/7	>2% over 3 months	3–8 weeks	Gabapentin, Pregabalin, Duloxetine;	Resolution in 3–12 months; 1 patient unresolved
Dabby, 2009 [35]	Case series	6	27–58 42 (mean)	5	1	0	0	6	3–5 yrs 4 yrs (mean)	Painful neuropathy	Diabetic retinopathy -2/6	Rapid correction of glycemic levels	2–4 weeks	Carbamazepine Gabapentinoids Duloxetine Amitriptyline Opioids	Symptoms resolution over 3–8 months
Tesfaye, 1996 [11]	Case series	5	34–64 48 (mean)	3	2	2	3	0	2.4 (2, 5, 5 years; 2 new-onset) (mean)	Painful neuropathy Autonomic dysfunction	None reported	Rapid insulin titration	4–8 weeks	Imipramine Codeine Symptomatic treatment	Significant improvement over 6–9 months
Koh, 2019 [47]	Case series	5	43–72 55 (mean)	3	2	0	5	0	6–20 yrs 10 (mean)	Painful neuropathy Autonomic dysfunction	Major acute systemic conditions requiring hospitalization	4.7% over 28–98 days	2–8 weeks	NR	NR
Tran, 2015 [48]	Case series	4	41–57 48 (mean)	3	1	2	2	0	1 month–5 yrs 1.25 yrs (mean)	Painful neuropathy Autonomic dysfunction	None reported	7.2% over several weeks	4–6 weeks	Duloxetine Pregabalin	Variable recovery after 12–14 months
Siddique, 2020 [32]	Case series	3	56–67 60 (mean)	3	0	0	3	0	8 months–2 yrs 1 yrs (Mean)	Painful neuropathy Lumbosacral radiculoplexus neuropathy OM nerve palsy	None reported	>2% over 3 months	Several weeks	Pregabalin Symptomatic management	Significant improvement of symptoms over 3–12 months
Van Haren, 2017 [37]	Case series	2	15–22 19 (mean)	0	2	2	0	0	Not mentioned	Painful neuropathy	Bilateral cataract-1/2 Proliferative retinopathy-1/2 Macular edema-1/2	Rapid correction of glycemic levels	Several weeks	Gabapentinoids Opioids Amitriptyline Duloxetine	Symptoms improvement over several months
Hwang, 2016 [49]	Case series	2	22–24 23 (mean)	0	2	2	0	0	16 yrs	Painful neuropathy Autonomic dysfunction	Diabetic steatohepatitis-1/2	>2% over 12 months	Several months	Gabapentin, Pregabalin, Fludrocortisone	Significant improvement after 1–9 months
Mudenha, 2016 [36]	Case series	2	28–30 29 (mean)	0	2	2	0	0	3–23 yrs 13 (mean)	Painful neuropathy	Pregnancy at symptoms onset-2/2	7.2% over 3 months, 4.6% over 7 weeks	2–3 weeks 2.5 (mean)	Amitriptyline Gabapentin Opioids	Complete recovery after 10 weeks in the first case Partial recovery after 6 months in the second case
Aladdin, 2017 [50]	Case series	2	43–46 (mean 44.5)	1	1	1	0	1	14–16 yrs 15 (mean)	Painful neuropathy Autonomic dysfunction	None reported	8.2% over 3 months (Ist case) NR (IInd case)	Several weeks 5 months	Pregabalin Gabapentin Opioids	Complete recovery after 2 years Partial resolution after 1 year
Yip, 2011 [57]	Case series	2	18–27 22.5 (mean)	0	2	2	0	0	new-onset; diagnosed at 16 (~11 years)	Painful neuropathy Hypertension-pseudo-phaeochromocytoma	Graves thyrotoxicosis-1/2	NR (Ist case) 7.4% (IInd case)	1 month	Beta-blockers Calcium channel blockers Ist-tramadol, diazepam and amitriptyline- 9 months IInd-amitriptyline, gabapentine-10 months	Complete recovery within one year
Schueller, 2025 [65]	Case report	1	50	0	1	0	1	0	Not mentioned	Painful neuropathy Lower limbs weakness Autonomic dysfunction	Hyperlipidemia Chronic iron-deficiency anemia	Abrupt drop of 7.9%	5 months	Gabapentin Duloxetine Glucose monitoring	Some improvement of symptoms
Varadharaju, 2019 [51]	Case report	1	16	0	1	1	0	0	0	Painful neuropathy Autonomic dysfunction	None reported	10.4% in 2 months	5 weeks	Amitriptyline, Pregabalin, Gabapentin	Complete resolution in 2 months
Hernandez, 2018 [41]	Case report	1	25	1	0	1	0	0	2	Painful neuropathy	None reported	6.2% in 8 weeks	2 weeks	Duloxetine Tramadol NSAID	Quasicomplete remission at 6 months
Abdelhamid **, 2023 [44]	Case report	1	60	1	0	0	1	0	0	Painful neuropathy	None reported	7.7% over 4 months	6 weeks	Pregabalin Duloxetine	Quasicomplete resolution after 1year
Hu, 2025 [66]	Case report	1	26	1	0	1	0	0	3	Painful neuropathy Perineal pain	None reported	2.7% over 2 months	2 months	Gabapentin, Pregabalin, Carbamazepine, Tramadol, Buprenorphine	Significant pain improvement at 5 months
Guisado Vasco, 2020 [52]	Case report	1	23	0	1	1	0	0	5	Painful neuropathy Autonomic dysfunction	None reported	3% over 2 months	Few weeks	Gabapentin Eslicarbazepine Tramadol	Significant improvement after 6 months
Rasmussen, 2022 [63]	Case report	1	16	0	1	1	0	0	0	Painful neuropathy Autonomic dysfunction Restless legs syndrome	None reported	9.8% over 3 months	1 month	Pregabalin, Gabapentin, Amitriptyline	Partial improvement after 18 months
Wilson, 2003 [38]	Case report	1	14	1	0	1	0	0	1	Painful neuropathy	None reported	6.5% over 9 weeks	3 months	Gabapentin, Hydrocodone, Phenytoin	Significant improvement after 3 months
Rothacker, 2014 [40]	Case report	1	20	1	0	1	0	0	5	Painful neuropathy	Persistent sinus tachycardia—possible diabetic neuropathy	Rapid insulin titration	Several weeks	Gabapentin Duloxetine Opioids	Significant improvement after 8 months
Oleynick, 2020 [53]	Case report	1	51	0	1	1	0	0	15	Aggravation of painful neuropathy Autonomic dysfunction	Ischemic cardiomyopathy Diabetic nephropathy Diabetic neuropathy Gastroparesis	5.1% over 6 months	2 weeks	Gabapentin, Pregabalin, Venlafaxine	Return to baseline in 2 weeks
Broadhead, 2021 [74]	Case report	1	66	0	1	0	1	0	9	Sensory-motor neuropathy	Hypertension Hyperlipidemia Obstructive sleep apnea	Glycemic correction over 3 months	Not mentioned	Relaxation of glycemic targets	Improvement after 3 weeks
Song, 2009 [39]	Case report	1	30	1	0	1	0	0	15	Painful neuropathy Autonomic dysfunction	None reported	2.2% over 4 weeks	1 week	Amitriptyline	Significant improvement after 1 month
Kim, 2020 [54]	Case report	1	24	0	1	1	0	0	10	Painful neuropathy Autonomic dysfunction	None reported	9.4% over 3 months	1 month	Pregabalin, Amitriptyline, Tapentadol	Complete remission after 1 month
Yuen, 2001 [58]	Case report	1	32	1	0	1	0	0	0	Painful neuropathy Myalgia Autonomic dysfunction	None reported	7.8% over 6 weeks	6 weeks	Symptomatic treatment Glycemic monitoring	Significant improvement over 6 months
Llewelyn, 1986 [12]	Case report	1	18	0	1	1	0	0	8	Painful neuropathy	Early diabetic retinopathy	9.5% over 6 weeks	Several weeks	Opioids Mianserin Chlorprothixene	Significant improvement after 24–26 weeks
Stainforth-Dubois, 2021 [55]	Case report	1	20	0	1	1	0	0	0	Painful neuropathy Autonomic dysfunction	None reported	10.3% in 3 months	3–6 months	Venlafaxine Symptomatic treatment	Significant improvement after 18 months
Ferreira, 2021 [43]	Case report	1	32	1	0	1	0	0	3	Painful neuropathy	No diabetic complications reported Active smoking Deep vein thrombosis	4.8% in 6 months	1 month	Amitriptyline	Complete resolution after 6 months
Chandler, 2020 [42]	Case report	1	18	1	0	1	0	0	0	Painful neuropathy	None reported	7.3% over 7 weeks	2 weeks	Gabapentin, Carbamazepine, Amitriptyline, Tramadol, Pregabalin	Gradual improvement over 18 months
Meillet, 2019 [60]	Case report	1	35	0	1	1	0	0	23	Painful neuropathy Ataxic gait Autonomic dysfunction	Pancreas islet transplant Immunosuppressive therapy Proliferative retinopathy	10% over 2 months	10 days	Pregabalin Duloxetine Venlafaxine Amitriptyline Opioids	No recovery after 6 years
Triplett, 2019 [61]	Case report	1	27	0	1	1	0	0	20	Painful neuropathy Multifocal weakness Autonomic dysfunction	Hypertension Hypothyroidism Diabetic retinopathy Diabetic nephropathy	7.6% over 6 weeks	Several days	Fludrocortisone Rehydration—NaCl Symptomatic treatment	No recovery after 8 weeks Lost to follow-up
Sahyouni, 2024 [45]	Case report	1	50	1	0	0	1	0	0	Painful neuropathy	Hyperthyroidism Obstructive sleep apnea Obesity	8% over 3 months	Several days	Topiramate Gabapentin	Partial recovery after 1 year
Zahir, 2025 [67]	Case report	1	37	0	1	0	1	0	NR	Painful neuropathy Asymmetric proximal weakness Autonomic dysfunction	Hypertension Chronic kidney disease Hypothyroidism Diabetic retinopathy	7% over 10 weeks	Several weeks	Complex pain regimen Opioids	No recovery after 6 months
Kanbour, 2023 [71]	Case report	1	36	1	0	1	0	0	0	Painful radiculopathy Restless leg syndrome	None reported	8.4% over 1 month	Several weeks	Gabapentin, Duloxetine, Nortriptyline, Tramadol, Fentanyl	Partial recovery after 1 year
Lee, 2025 [72]	Case report	1	25	1	0	1	0	0	16	2 TIND episodes: I. Painful neuropathy (2019) II. Painful radiculopathy (2023)	Peripheral neuropathy Asthma Chronic pancreatitis Vitamin D deficiency	4.1% over 10 months (Ist case), 8% over 7 months (IInd case)	Several months	Gabapentinoids Duloxetine Nortriptyline Topical lidocaine Opioids Lumbar sympathetic block	Ist episode-recovery over 1 year IInd episode-partial recovery after 1 year
MacIsaac, 2026 [56]	Case report	1	51	1	0	0	1	0	NR	Painful neuropathy Autonomic dysfunction	Peripheral neuropathy Bilateral cataracts	9.4% over 3 months	3 weeks	IV fluids Fludrocortisone, midodrine, Caffeine Domperidone, Pregabalin	Partial recovery after 6 months
Aslam, 2014 [70]	Case report	1	31	1	0	1	0	0	0	Right-side lower abdominal painful neuropathy Weight loss	None reported	7.3% over 2 months	2 months	Amitriptyline	Partial recovery after 1 month
Partridge, 2009 [73]	Case report	1	19	0	1	1	0	0	4	Autonomic dysfunction Weight loss	None reported	5.8% over 6 months	NR	Supportive treatment Prokinetic Ciprofloxacin Metronidazole	Complete recovery
Deorchis, 2013 [59]	Case report	1	16	0	1	1	0	0	2	Painful distal ascending neuropathy Weight loss	Psychiatric comorbidity—bipolar disorder, depression	7.2%	Subacute	Pregabalin NSAID Amitriptyline	Partial recovery after 6 months
Pereira, 2023 [64]	Case report	1	40	0	1	0	1	0	12	Painful neuropathy Low back pain Asymmetric lower limbs weakness; proximal atrophy Autonomic dysfunction	Pregnancy at 8 weeks gestation	6.1% over 4 weeks	Rapid progression	Pain symptomatic treatment Intensive motor physical therapy	Partial recovery after 10 weeks
Shimizu, 2019 [62]	Case report	1	28	0	1	1	0	0	18	Painful neuropathy Lower limbs weakness Gait disturbance	Diabetic nephropathy Proliferative retinopathy	7% over 2 months	2 months	Acetaminophen Mexiletine hydrochloride	Partial recovery after 1 month
Abohalima, 2025 [68]	Case report	1	17	1	0	1	0	0	1 month	Autonomic dysfunction Painful ascending neuropathy Distal lower limbs weakness	None reported	5.1% over 3 months	1 month	Domperidone immune globulin intravenous—no response Gabapentin, Nortriptyline, Opioids- morphine	NR
Ndip, 2009 [69]	Case report	1	31	0	1	1	0	0	28	Thoracic neuropathy—severe pain on the left upper quadrant and left flank-T8–T12	End-stage renal disease Simultaneous kidney–pancreas transplants 5 years before- Tacrolimus	Second pancreatic transplant-> 4.9% over 3 months	NR	Pregabalin, Amitriptyline	Complete recovery several weeks later No complaints after 16 months follow-up
Teixeira, 2025 [33]	Case report	1	59	1	0	0	1	0	20	Intermittent burning paresthesias throughout his body; paraesthesia had become painful, severe, and localized but symmetrical in his thighs Weight loss	Hypertension	12.5% in 3 months	2 weeks	Supportive treatment	At 1 year after discharge, he could walk long distances with one aid, and his muscle strength and thigh muscle atrophy had improved
Total	13 case series + 33 case reports + 1 case–control	219	-	91	128	131	81	7	-	-		-	-	-	-

M, male; F, female; yrs, years; T1DM, type 1 diabetes; T2DM, type 2 diabetes; NR, not reported. * The type of diabetes was recorded as specified in the source publications. Dabby et al. [35] did not specify the type of diabetes for any of the six patients (three were treated with insulin, and three without), and Aladdin et al. [50] did not specify it for one of their two patients; these seven patients are listed as “NR” (not reported/not specified) and were not assigned to any of the categories. ** Abdelhamid et al. [44] described latent autoimmune diabetes in adults, listed here as T2DM.
