# Supplementary material for: Treatment-Induced Neuropathy of Diabetes: Clinical Insights from a Case Series and Systematic Review
Source: J Clin Med. 2026 Nov 9;15(21):8760. doi: 10.3390/jcm15218760 (PMC13764111; doi:10.3390/jcm15218760)
Supplement: Supplementary file 1 [file jcm-15-08760-s001.zip › Table_S1_PRISMA_2020_checklist_corrected.pdf]

Supplementary Materials

Treatment-Induced Neuropathy of Diabetes: Clinical Insights from a Case Series and Systematic Review

Table S1a. PRISMA 2020 checklist.

Checklist items are abbreviated for readability; item numbering follows the PRISMA 2020 statement. Section numbers refer to the main manuscript.

Section and Topic	Item #	Checklist item (abbreviated)	Location where item is reported
**TITLE**			
Title	1	The report is identified as a systematic review.	Title page — the title states "…Case Series and Systematic Review".
**ABSTRACT**			
Abstract	2	A structured abstract is provided, following the PRISMA 2020 for Abstracts checklist.	Abstract, p. 1; item-by-item mapping in Table S1b below.
**INTRODUCTION**			
Rationale	3	The rationale for the review is described in the context of existing knowledge.	Section 1, paragraphs 2–6.
Objectives	4	An explicit statement of the objective(s) or question(s) the review addresses.	Section 1, final paragraph (primary and secondary objectives); Section 2.2, paragraph 3 (population–exposure–outcome [PEO] review question).
**METHODS**			
Eligibility criteria	5	Inclusion and exclusion criteria are specified, together with how studies were grouped for the synthesis.	Section 2.2, paragraph 4 (inclusion and exclusion criteria, the two observational studies, and the rationale for including only the lumbosacral radiculoplexus patients of the case–control study); studies grouped for synthesis by clinical phenotype (Table 2, Section 4.1).
Information sources	6	All databases, registers and other sources searched are specified, with the date each was last searched.	Section 2.2, paragraph 2 (PubMed, Scopus, Web of Science and Google Scholar, 1980–2026; date of last search 9 August 2026); full detail in Table S2. All included studies were identified through these database searches.
Search strategy	7	The full search strategy for each source is presented, including any filters and limits.	Table S2 — complete search strings as run in each database, with filters and limits (year limit 1980–2026; language restriction applied at screening). Differences in sensitivity between databases are stated in Table S2.
Selection process	8	The methods used to decide whether a study met the inclusion criteria are described, including how many reviewers screened each record and whether they worked independently.	Section 4.1, paragraph 2 — records screened independently by two investigators, disagreements resolved by consensus; Figure 1.
Data collection process	9	The methods used to collect data from reports are described, including the	Section 2.2, paragraph 5 — extraction by one investigator into a pre-specified template; all extracted variables

Section and Topic	Item #	Checklist item (abbreviated)	Location where item is reported
		number of reviewers and how they worked.	independently verified by a second investigator, discrepancies resolved by consensus; the AI-assisted verification step is described in Section 2.2 and in the AI-use statement.
Data items	10a	All outcomes for which data were sought are listed and defined.	Section 2.2, paragraphs 3 and 5 (outcomes and extracted variables); Table 2 and Table A1.
Data items	10b	All other variables for which data were sought are listed and defined, with any assumptions made about missing information.	Section 2.2, paragraph 5 — other variables extracted; missing information recorded as "NR" (not reported) in Tables 2 and A1; diabetes type recorded as stated in the source and not inferred.
Study risk of bias assessment	11	The methods used to assess risk of bias in the included studies are described, including the tool used and the number of reviewers.	Section 2.2, paragraph 2 — methodological quality assessed with the tool of Murad et al. (8 items, 4 domains), with an overall rating based on three items (exposure, outcome, exclusion of alternative causes) and no summed score; a draft assessment prepared with large language model assistance was verified and corrected by two investigators; item-level ratings in Table S3.
Effect measures	12	For each outcome, the effect measure used in the synthesis or presentation of results is specified.	Section 2.2, paragraph 5 — no effect measures; results summarized descriptively (counts, proportions, medians, ranges).
Synthesis methods	13a	The process used to decide which studies were eligible for each synthesis is described.	Section 2.2, paragraphs 4–5 — all eligible publications contributed to a single descriptive synthesis, grouped by clinical phenotype (Table 2).
Synthesis methods	13b	Any methods required to prepare the data for presentation or synthesis are described.	Section 2.2, paragraph 5 — data extracted into a common template; individual patient data reported as ranges and summary statistics as published; no data conversion or imputation.
Synthesis methods	13c	The methods used to tabulate or visually display the results of individual studies and syntheses are described.	Table 2 (grouped by phenotype), Table A1 (case level) and Section 4.1.
Synthesis methods	13d	The methods used to synthesise results are described, with rationale.	Section 2.2, paragraph 5 — descriptive synthesis only; no inferential statistics or meta-analysis because of heterogeneity and the case-report nature of the evidence; see Limitations (Section 5).
Synthesis methods	13e	Any methods used to explore possible causes of heterogeneity are described.	Not applicable — no quantitative synthesis. Heterogeneity is described narratively in Section 4.1 and in the Limitations (Section 5).
Synthesis methods	13f	Any sensitivity analyses conducted to assess robustness of the synthesised results are described.	Section 2.2, paragraph 5 — sensitivity analysis recalculating descriptive totals after excluding the potentially overlapping

Section and Topic	Item #	Checklist item (abbreviated)	Location where item is reported
			cohort of Gibbons and Freeman (2010) [22]; results in Section 4.1, paragraph 3.
Reporting bias assessment	14	Any methods used to assess risk of bias due to missing results are described.	Not formally assessed (funnel-plot methods are not applicable to case reports and case series); publication and reporting bias are discussed in the Limitations (Section 5).
Certainty assessment	15	Any methods used to assess certainty (or confidence) in the body of evidence are described.	Not performed. GRADE was not applied because all evidence is uncontrolled case-level data (very low certainty by design); the resulting limitations are stated in Section 5.
**RESULTS**			
Study selection	16a	The numbers of records screened, assessed for eligibility and included are reported, ideally using a flow diagram.	Section 4.1, paragraphs 1–2; Figure 1.
Study selection	16b	Studies that appeared to meet the inclusion criteria but were excluded are cited, with reasons.	Figure 1 — 44 full-text articles excluded. CONFIRM that Figure 1 lists the reasons with numbers; if not, add a supplementary list of the 44 excluded articles with reasons and cite it here.
Study characteristics	17	Each included study is cited and its characteristics presented.	Table 2 (all 47 included publications) and Table A1; summarized in Section 4.1.
Risk of bias in studies	18	Risk of bias assessments are presented for each included study.	Table S3 — item-level ratings for each publication; summary in Section 4.1, paragraph 6.
Results of individual studies	19	For all outcomes, the summary statistics for each study are presented.	Table 2 and Table A1 — study-level and case-level data for every included report.
Results of syntheses	20a	For each synthesis, the characteristics and risk of bias of the contributing studies are briefly summarised.	Section 4.1, paragraphs 3–6 — characteristics and methodological quality of the contributing publications.
Results of syntheses	20b	The results of all statistical syntheses are presented.	Not applicable — no meta-analysis. Descriptive results are reported in Section 4.1.
Results of syntheses	20c	The results of any investigations of possible causes of heterogeneity are presented.	Not applicable — no quantitative synthesis. Sources of heterogeneity are discussed in Section 4.1 and in the Limitations (Section 5).
Results of syntheses	20d	The results of any sensitivity analyses are presented.	Section 4.1, paragraph 3 — after excluding the potentially overlapping Gibbons and Freeman (2010) cohort [22], descriptive proportions differed by less than 1 percentage point from the full series.
Reporting biases	21	Risk of bias due to missing results is presented for each synthesis.	Not formally assessed — see item 14; publication bias is discussed in the Limitations (Section 5).
Certainty of evidence	22	Assessments of certainty in the body of evidence are presented for each outcome.	Not formally assessed — see item 15; the limitations of case-level evidence are acknowledged in Section 5.

Section and Topic	Item #	Checklist item (abbreviated)	Location where item is reported
**DISCUSSION**			
Discussion	23a	A general interpretation of the results in the context of other evidence is provided.	Section 5, paragraphs 1–6.
Discussion	23b	Limitations of the evidence included in the review are discussed.	Section 5, Limitations (final two paragraphs).
Discussion	23c	Limitations of the review processes used are discussed.	Section 5, Limitations — language restriction, search sources, retrospective registration, methodological appraisal with large language model–assisted steps, possible overlap between two cohorts from the same centre, absence of quantitative synthesis.
Discussion	23d	The implications of the results for practice, policy and future research are discussed.	Section 5, paragraph 7 (risk-adapted approach, Figure 2); Section 6.
**OTHER INFORMATION**			
Registration and protocol	24a	The review is registered, including the register name and registration number, or it is stated that the review was not registered.	Section 2.2, paragraph 1 — protocol registered retrospectively in the Open Science Framework Registries on 18 August 2026, after the literature search (last search 9 August 2026) and study selection were completed [28]; https://osf.io/r2da4.
Registration and protocol	24b	Where the review protocol can be accessed is indicated, or it is stated that a protocol was not prepared.	Reference [28]; https://osf.io/r2da4.
Registration and protocol	24c	Amendments to the information provided at registration or in the protocol are described and explained.	Section 2.2, paragraph 6 — four changes relative to the registered protocol are listed and explained: review question reframed from PICO to PEO; sensitivity analysis excluding a potentially overlapping cohort added; diabetes type recorded as "not reported" when not stated; Murad et al. tool used instead of the Cochrane risk-of-bias tool.
Support	25	Sources of financial or non-financial support for the review, and the role of the funders, are described.	Funding statement — this research received no external funding.
Competing interests	26	Competing interests of the review authors are declared.	Conflicts of Interest statement.
Availability of data, code and other materials	27	It is stated which of the following are publicly available and where: template data collection forms; extracted data; analytic code; other materials.	Data Availability Statement. Extracted data for all included studies are presented in Table 2, Table A1 and Table S3; the data-extraction template and other materials are available from the corresponding author on reasonable request. No analytic code was generated.

Table S1b. PRISMA 2020 for Abstracts checklist.

Section numbers refer to the main manuscript.

Section and Topic	Item #	Checklist item (abbreviated)	Location where item is reported
Title	1	The report is identified as a systematic review.	Title.
Objectives	2	The objective(s) or question(s) the review addresses are given.	Abstract — "Background/Objectives".
Eligibility criteria	3	The inclusion and exclusion criteria for the review are specified.	Abstract — "Methods".
Information sources	4	The information sources searched and the date of the last search are specified.	Abstract — "Methods" (databases named); the date of the last search (9 August 2026) is given in Section 2.2 and Table S2.
Risk of bias	5	The methods used to assess risk of bias in the included studies are specified.	Not reported in the abstract (200-word limit). Methodological quality was assessed with the tool of Murad et al. — see Section 2.2 and Table S1a, item 11.
Synthesis of results	6	The methods used to present and synthesise results are specified.	Abstract — "Methods" ("Data were synthesized descriptively").
Included studies	7	The total number of included studies and participants, and relevant study characteristics, are given.	Abstract — "Results" (47 publications, 219 patients).
Synthesis of results	8	Results for the main outcomes are presented, ideally indicating the number of studies and participants for each.	Abstract — "Results".
Limitations of evidence	9	Limitations of the evidence included in the review are given.	Abstract — "Results"/"Conclusions"; full statement in Section 5 (Limitations).
Interpretation	10	A general interpretation of the results and important implications is provided.	Abstract — "Conclusions".
Funding	11	The primary source of funding for the review is given.	Not reported in the abstract (journal word limit); see the Funding statement — no external funding.
Registration	12	The register name and registration number are given.	Not reported in the abstract (word limit). Registered retrospectively in the Open Science Framework Registries, https://osf.io/r2da4 — see Section 2.2 and Table S1a, item 24a.

Reference: Page MJ, McKenzie JE, Bossuyt PM, et al. The PRISMA 2020 statement: an updated guideline for reporting systematic reviews. BMJ 2021;372:n71. doi:10.1136/bmj.n71
