# Supplementary material for: Treatment-Induced Neuropathy of Diabetes: Clinical Insights from a Case Series and Systematic Review
Source: J Clin Med. 2026 Nov 9;15(21):8760. doi: 10.3390/jcm15218760 (PMC13764111; doi:10.3390/jcm15218760)
Supplement: Supplementary file 1 [file jcm-15-08760-s001.zip › Table_S2_search_strategy_new.pdf]

Supplementary Materials

Treatment-Induced Neuropathy of Diabetes: Clinical Insights from a Case Series and Systematic Review

Table S2. Full electronic search strategy, by database.

No language filter was applied within the search strings; the restriction to publications in English or French was applied at the title and abstract screening stage. No additional filters or limits were used beyond the publication-year limits shown.

Database	Interface and coverage	Search string as run	Date last searched	Records retrieved
PubMed	NCBI PubMed; limited to 1980–2026	("Diabetic Neuropathies"[Mesh] OR "diabetic neuropathy"[tiab]) AND ("treatment-induced neuropathy of diabetes"[tiab] OR "TIND"[tiab] OR "insulin neuritis"[tiab] OR "insulin-induced neuropathy"[tiab] OR "acute painful neuropathy"[tiab]) AND ("1980"[Date - Publication] : "2026"[Date - Publication])	9 August 2026	216
Scopus	Elsevier Scopus; 1980–2026	TITLE-ABS-KEY ("treatment-induced neuropathy of diabetes" OR "TIND" OR "insulin neuritis" OR "insulin-induced neuropathy" OR "acute painful neuropathy after glycemic correction") AND PUBYEAR > 1979 AND PUBYEAR < 2027	9 August 2026	644
Web of Science	Clarivate Web of Science Core Collection; 1980–2026	TS = ("treatment-induced neuropathy of diabetes" OR "TIND" OR "insulin neuritis" OR "insulin-induced neuropathy" OR "acute painful neuropathy after glycemic correction"), Timespan 1980–2026	9 August 2026	541
Google Scholar	Google Scholar; 1980–2026; the first 18 pages of results (180 records) were screened	"treatment-induced neuropathy of diabetes" OR "insulin neuritis" OR "insulin-induced neuropathy" OR "acute painful neuropathy after glycemic correction"	9 August 2026	180
**Total records identified**				**1581**
**Duplicate records removed**				**577**
**Records after removal of duplicates**				**1004**

Records were exported to a reference manager and de-duplicated before title and abstract screening. Screening and full-text assessment were performed independently by two investigators, with disagreements resolved by consensus. All included studies were identified through the database searches shown above; no additional eligible reports were identified by other methods.

The search string run in PubMed combined a population term (diabetic neuropathy) with the exposure terms and included the broader phrase "acute painful neuropathy". The strings run in Scopus, Web of Science and Google Scholar used the exposure terms alone. Sensitivity therefore differed between databases.
