# Supplementary material for: Treatment-Induced Neuropathy of Diabetes: Clinical Insights from a Case Series and Systematic Review
Source: J Clin Med. 2026 Nov 9;15(21):8760. doi: 10.3390/jcm15218760 (PMC13764111; doi:10.3390/jcm15218760)
Supplement: Supplementary file 1 [file jcm-15-08760-s001.zip › Table_S3_Supplementary.pdf]

Table S3. Methodological quality of the included publications, assessed with the tool of Murad et al., and classification according to the Gibbons–Freeman diagnostic criteria.

#	Study [ref.]	Design	N	Q1	Q2	Q3	Q4	Q5	Q6	Q7	Q8	Overall	Gibbons–Freeman criteria
1	Gibbons & Freeman 2015 [8]	Retrospective cohort	104	Yes	Yes	Partial	Yes	No	Yes	Yes	Yes	Moderate	Met
2	Triplett 2025 [31]	Case-control	26	Yes	Yes	Yes	Yes	Yes	Partial	No	Partial	Good	Onset beyond 8 weeks
3	Gibbons & Freeman 2010 [22]	Case series	16	Yes	Yes	Yes	Yes	No	No	Yes	Yes	Good	Met
4	Alexandrou 2020 [46]	Case series	7	No	Yes	No	Partial	No	No	Yes	Yes	Poor	Met
5	Dabby 2009 [35]	Case series	6	Unclear	Yes	Partial	No	No	No	Yes	Partial	Poor	Insufficient data
6	Tesfaye 1996 [11]	Case series	5	Unclear	Partial	Yes	No	No	No	Yes	Partial	Poor	Met
7	Koh 2019 [47]	Case series	5	Yes	Yes	Partial	Yes	No	No	No	Partial	Moderate	Met
8	Tran 2015 [48]	Case series	4	Yes	Yes	Partial	Partial	No	No	Yes	Yes	Poor	Met in some patients only
9	Siddique 2020 [32]	Case series	3	Unclear	Partial	Partial	Partial	No	No	Partial	Yes	Poor	Met in some patients only
10	van Haren 2017 [37]	Case series	2	Unclear	Partial	Partial	No	No	NA	Yes	Partial	Poor	Insufficient data
11	Hwang 2016 [49]	Case series	2	Unclear	Partial	Partial	No	No	NA	Partial	Partial	Poor	Met in some patients only
12	Mudenha 2016 [36]	Case series	2	Unclear	Yes	No	Partial	No	NA	Yes	Yes	Poor	Met
13	Aladdin 2017 [50]	Case series	2	Unclear	Yes	Yes	No	Yes	No	Yes	Partial	Moderate	Insufficient data
14	Yip 2011 [57]	Case series	2	Unclear	Partial	Yes	Yes	No	NA	Yes	Yes	Moderate	Insufficient data
15	Schueller 2025 [65]	Case report	1	Unclear	Yes	Yes	Yes	No	NA	Partial	Yes	Good	Onset beyond 8 weeks
16	Varadharaju 2019 [51]	Case report	1	Unclear	Yes	Yes	Yes	Yes	NA	Yes	Yes	Good	Met
17	Hernandez 2018 [41]	Case report	1	Unclear	Yes	Yes	Yes	No	NA	Yes	Yes	Good	Met
18	Abdelhamid 2023 [44]	Case report	1	Unclear	Yes	Yes	Partial	No	NA	Yes	Yes	Moderate	HbA1c criterion met; onset interval unclear
19	Hu 2025 [66]	Case report	1	Unclear	Yes	Yes	Partial	No	NA	Partial	Yes	Moderate	HbA1c criterion met; onset interval unclear
20	Guisado Vasco 2020 [52]	Case report	1	Unclear	Yes	Yes	Yes	No	NA	Yes	Yes	Good	Met
21	Rasmussen 2022 [63]	Case report	1	Unclear	Yes	Yes	No	No	NA	Yes	Yes	Moderate	Met
22	Wilson 2003 [38]	Case report	1	Unclear	Yes	Yes	Partial	Yes	NA	Partial	Yes	Moderate	HbA1c criterion met; onset interval unclear
23	Rothacker 2014 [40]	Case report	1	Unclear	Partial	Partial	No	No	NA	Yes	Partial	Poor	Insufficient data
24	Oleynick 2020 [53]	Case report	1	Unclear	Yes	Partial	Yes	No	NA	Yes	Yes	Moderate	Met
25	Broadhead 2021 [74]	Case report	1	Unclear	Partial	Partial	Partial	No	NA	No	Partial	Poor	Insufficient data
26	Song 2009 [39]	Case report	1	Unclear	Yes	Yes	No	No	NA	Partial	Yes	Moderate	Met
27	Kim 2020 [54]	Case report	1	Unclear	Yes	Yes	No	No	NA	Yes	Yes	Moderate	Met
28	Yuen 2001 [58]	Case report	1	Unclear	Yes	Yes	Yes	No	NA	Yes	Yes	Good	Met
29	Llewelyn 1986 [12]	Case report	1	Unclear	Yes	Yes	Yes	No	NA	Yes	Yes	Good	Met
30	Stainforth-Dubois 2021 [55]	Case report	1	Unclear	Yes	Yes	Yes	No	NA	Yes	Yes	Good	HbA1c criterion met; onset interval unclear
31	Ferreira 2021 [43]	Case report	1	Unclear	Yes	Yes	Partial	No	NA	Yes	Yes	Moderate	Met
32	Chandler 2020 [42]	Case report	1	Unclear	Yes	Yes	No	No	NA	Yes	Yes	Moderate	Met
33	Meillet 2019 [60]	Case report	1	Unclear	Yes	Partial	Yes	No	NA	Yes	Yes	Moderate	Met

#	Study [ref.]	Design	N	Q1	Q2	Q3	Q4	Q5	Q6	Q7	Q8	Overall	Gibbons–Freeman criteria
34	Triplett 2019 [61]	Case report	1	Unclear	Yes	Yes	Partial	No	NA	Partial	Yes	Moderate	Met
35	Sahyouni 2024 [45]	Case report	1	Unclear	Yes	Yes	Yes	No	NA	Yes	Yes	Good	Met
36	Zahir 2025 [67]	Case report	1	Unclear	Yes	Yes	Partial	No	NA	Yes	Yes	Moderate	HbA1c criterion met; onset interval unclear
37	Kanbour 2023 [71]	Case report	1	Unclear	Yes	Yes	Partial	No	NA	Yes	Yes	Moderate	Met
38	Lee 2025 [72]	Case report	1	Unclear	Yes	Yes	No	Yes	NA	Yes	Yes	Moderate	Onset beyond 8 weeks
39	MacIsaac 2026 [56]	Case report	1	Unclear	Yes	Yes	Yes	No	NA	Yes	Yes	Good	Met
40	Aslam 2014 [70]	Case report	1	Unclear	Yes	Partial	Partial	No	NA	Partial	Yes	Poor	HbA1c criterion met; onset interval unclear
41	Partridge 2009 [73]	Case report	1	Unclear	Yes	Partial	Yes	No	NA	Yes	Partial	Moderate	Insufficient data
42	Deorchis 2013 [59]	Case report	1	Unclear	Partial	Yes	Yes	No	NA	Yes	Yes	Moderate	Insufficient data
43	Pereira 2023 [64]	Case report	1	Unclear	Yes	Yes	Yes	No	NA	Partial	Yes	Good	Met
44	Shimizu 2019 [62]	Case report	1	Unclear	Yes	Yes	Yes	No	NA	Partial	Yes	Good	Met
45	Abohalima 2025 [68]	Case report	1	Unclear	Yes	Yes	Yes	No	NA	No	Partial	Good	Met
46	Ndip 2009 [69]	Case report	1	Unclear	Yes	Yes	Partial	No	NA	Yes	Partial	Moderate	HbA1c criterion met; onset interval unclear
47	Teixeira 2025 [33]	Case report	1	NA	Partial	NR	NR	NR	NA	NR	NR	Not assessed	HbA1c criterion met; onset interval unclear

Tool: Murad, M.H.; Sultan, S.; Haffar, S.; Bazerbachi, F. Methodological quality and synthesis of case series and case reports. BMJ Evid. Based Med. 2018, 23, 60–63. Items and operational rules agreed a priori:

Q1 Selection – Does the patient(s) represent the whole experience of the investigator (centre)? Yes, series collected over a defined period or consecutive patients; No, series explicitly assembled non-systematically; Unclear, single case reports and series without a defined period.

Q2 Exposure – Was the exposure adequately ascertained? Yes, HbA1c (or glucose) reported before and after correction, with the time interval; Partial, values or interval incomplete.

Q3 Outcome – Was the outcome adequately ascertained? Yes, neuropathy documented clinically and by at least one objective test (nerve conduction studies/electromyography, skin biopsy, autonomic or sudomotor testing); Partial, clinical diagnosis only, or objective testing in some patients only; No, no objective documentation.

Q4 Alternative causes – Were other alternative causes that may explain the observation ruled out? Yes, investigations for other causes of neuropathy reported; Partial, limited or non-specific work-up, or a competing cause present; No, not reported.

Q5 Challenge/rechallenge – Was there a challenge/rechallenge phenomenon? Yes, improvement after relaxation of glycemic targets and/or recurrence with a new rapid glycemic correction; No, not reported.

Q6 Dose–response – Was there a dose–response effect? Yes, relation between HbA1c fall and severity analysed; NA, not applicable (single case reports).

Q7 Follow-up – Was follow-up long enough for outcomes to occur? Yes, ≥6 months or until documented resolution; Partial, shorter; No, outcome not reported.

Q8 Reporting – Is the case(s) described with sufficient details? Yes, age, sex, diabetes type, HbA1c values with interval, onset timing, management and outcome all reported; Partial, some of these missing.

Overall judgement (no summed score), based on the items most relevant to diagnostic certainty (Q2, Q3, Q4): Good, all three Yes; Moderate, two Yes; Poor, one or none Yes; Not assessed, full text unavailable.

Gibbons–Freeman criteria [8]: HbA1c fall ≥2 percentage points per 3 months (applied proportionally over longer intervals) and onset within 8 weeks of the documented fall.

Assessment: a draft prepared with the assistance of a large language model was independently verified and corrected by two investigators (N.C.D., R.M.); agreement was 94% (353/376 items; Cohen's κ = 0.92), and disagreements were resolved by consensus. NA, not applicable; NR, not reported (full text unavailable).
